# Soil properties and rhizosphere interactions affecting nitrous oxide emissions with mitigation by nitrification inhibitors in rice growth stages

**DOI:** 10.3389/fpls.2025.1501410

**Published:** 2025-02-21

**Authors:** Haipeng Zhang, Yiyin Lu, Wanyi Li, Fuxing Liao, Juanjuan Wang, Hongcheng Zhang, Yanju Yang

**Affiliations:** Co-Innovation Center for Modern Production Technology of Grain Crops/Key Laboratory of Cultivated Land Quality Monitoring and Evaluation (Yangzhou University), Ministry of Agriculture and Rural Affairs, Research Institute of Rice Industrial Engineering Technology, Yangzhou University, Yangzhou, China

**Keywords:** nitrous oxide emissions, rice growth, nitrifying communities, nitrification inhibitors, rhizosphere soil

## Abstract

Nitrous oxide (N_2_O) emissions from paddy soils, particularly from the rice rhizosphere, significantly contribute to agricultural greenhouse gas outputs. This study explores N_2_O emission dynamics in rhizosphere (R) and non-rhizosphere (NR) soils from two distinct paddy types (JR and YC) during the primary rice growth stages (tillering, jointing, heading, and grain-filling). Cumulative N_2_O emissions were measured at 688.56, 762.90, 831.20, and 1072.32 µg N kg^-1^ for JR-NR, JR-R, YC-NR, and YC-R, respectively. Notably, JR-R and YC-R exhibited increases in cumulative N_2_O emissions by up to 20.04% and 28.23%, respectively, compared to their NR counterparts at different growth stages. These enhanced emissions were primarily associated with microbial genera *Nitrosospira* and *Nitrosospirae*, and influenced by factors such as electrical conductivity (EC) and available potassium (AK). The soil organic carbon to total nitrogen ratio (C/N) was a key determinant influencing *Nitrosospira* abundance. Additionally, nitrification inhibitors (NIs) demonstrated a substantial reduction in N_2_O emissions, with a decrease of 92.37% in JR-R and 91.93% in YC-R at selected growth stages, showing more pronounced effects compared to NR soils. These findings highlight the efficacy of NIs in significantly mitigating N_2_O emissions, particularly in rhizosphere soils. Variations in the efficiency of NIs across different soil types and growth stages suggest that optimizing application timing and developing tailored soil-specific strategies could further enhance the effectiveness of NIs in mitigating N_2_O emissions from paddy fields. This research provides essential insights for developing targeted mitigation strategies to reduce N_2_O emissions in rice cultivation and contributes to sustainable agricultural practices.

## 1 Introduction

Rice is the main staple cereals in China, and nitrogen is a crucial nutrient for the growth and development of rice. However, only 30%-40% applied nitrogen fertilizers are used by rice plant (Dong et al., 2022; Zhang et al., 2023), less efficiently in rice ecosystem than another major food-producing ecosystem (Su et al., 2021; Zhou, 2023). N_2_O is a significant byproduct of nitrogen fertilizer loss in paddy fields (Song et al., 2021). In China, it is estimated that N_2_O emissions from paddy fields account for approximately 7%-11% of the total N_2_O emissions from farmland (Zhou et al., 2019). N_2_O atmospheric levels are rising by approximately 0.73 ppb each year, driven by the growing global demand for food linked to population increases (Kimani et al., 2022). Consequently, it is crucial to effectively curtail N_2_O emissions from paddy fields. Understanding the factors that influence N_2_O emissions is essential for sustaining rice production and mitigating climate change impacts.

Nitrification and denitrification are the two primary microbial processes that influence N_2_O production in paddy soils. Water management is a critical factor in both processes, as it directly affects the oxygen concentration in the soil. Rice paddies were once considered insignificant sources of atmospheric N_2_O because under flooded anaerobic conditions, N_2_O, as an intermediate product of denitrification, would be further reduced to N_2_ (Cai et al., 2012). However, it is now widely accepted that rice plants, due to their aeration tissues, can transport oxygen to the rice rhizosphere, thereby creating conditions where nitrification can occur even in flooded paddy fields (Yan et al., 2000b; Yang et al., 2016). Recent studies have shown that the nitrification microbial communities in the rice rhizosphere differ significantly from those in bulk soil, which is a key factor influencing N_2_O emissions (Qi et al., 2023; Li et al., 2019). Moreover, rhizosphere soil properties such as soluble carbon content, pH, and the concentrations of ammonium nitrogen (NH_4_^+^-N) and nitrate nitrogen (NO_3_^-^-N) significantly affect N_2_O production in both the nitrification and denitrification processes (Ibrahim et al., 2020; Li et al., 2019; Qi et al., 2023; Zhong et al., 2022). Zhang et al. (2023) observed that rice root activity could reduce N_2_O emissions from the denitrification process, mainly due to the concentration of NO_3_^-^-N. When the NO_3_^-^-N supply is low, the roots compete with denitrifying microorganisms, reducing the denitrification process. Therefore, the rice rhizosphere is a dynamic and complex site where soil, rice roots, and microbes interact to influence N_2_O production.

The growth stage of rice plays a crucial role in directly affecting rhizosphere N_2_O emissions by modulating oxygen availability for the nitrification process, thus increasing N_2_O production from nitrification. Conversely, aerobic conditions can inhibit the denitrification process. Additionally, rice growth stages indirectly influence rhizosphere N_2_O emissions by altering the production and composition of root exudates, such as soluble carbon, which serves as an energy source for denitrification. These exudates also affect rhizosphere soil pH, a critical variable that influences both nitrification and denitrification processes. For example, Shaaban et al. (2023) demonstrated that as pH increases, the molar fraction of N_2_O decreases during denitrification. Thus, both the rice rhizosphere zone and the rice growth stage are pivotal factors in modulating N_2_O production in paddy fields.

Compared to the extensive research on factors and mitigation strategies for controlling N_2_O emissions in paddy fields, investigations specifically focusing on the factors influencing N_2_O production within the rice rhizosphere remain limited. Previous efforts to mitigate N_2_O emissions in paddy fields have primarily involved strategies such as the application of nitrification inhibitors (NIs), biochar, water management practices, and slow-release fertilizers. These approaches can reduce N_2_O emissions and improve nitrogen use efficiency by limiting the availability of substrates for microbial nitrification and denitrification processes (Butterbach-Bahl et al., 2013). NIs, for instance, have been reported to reduce N_2_O emissions by 11%–96%, although in certain cases, no significant effect has been observed (Zhong et al., 2022; Butterbach-Bahl et al., 2013). The observed variability in these results across different regions can be attributed to factors such as differences in rice varieties, levels of N fertilizer application, and the soil properties of the paddies. Moreover, the timing and location of NIs application further complicate efforts to consistently reduce N_2_O emissions in rice paddy systems.

In this study, we carried out pot and laboratory incubation experiments to assess N_2_O emissions in both R and NR soils at key stages of rice growth. The objectives of these experiments are: (1) to quantify the variations in N_2_O emissions in rhizosphere and non-rhizosphere paddy soil; (2) to identify the differences in N_2_O flux among various growth stages within the same rice soil; (3) to evaluation the primary soil abiotic and biotic factors that influence N_2_O emissions in soils and the impact of NIs on reducing N_2_O emissions in these specific soil zones.

## 2 Materials and methods

### 2.1 Materials

The soils tested were sampled from two locations: Jurong, Jiangsu Province, China (JR, 28°15’N, 116°55’E), where the acidic paddy soil has a clay texture and belongs to the Lower Shu Loess; and Yancheng (YC, 33°43’N, 118°86’E), where the alkaline paddy soil has a loam texture and is formed from lake sediment. Both areas use a cropping system that involves rotating dryland and paddy fields. In March in 2020, fresh topsoil samples (0 - 20 cm) were collected from both locations using the *S*-shape sampling method. The samples were thoroughly mixed, and any stones, roots, and crop residues were removed. Some of the soil samples were air-dried and used to determine the soil’s basic physical and chemical properties, while the rest were used for pot experiments. **Table 1** lists the basic physical and chemical properties of the soil. The experiment used the rice variety Nanjing 9108, and the rice seeds were provided by the Jiangsu Academy of Agricultural Sciences.

**Table 1:** Location, pH, nitrate nitrogen (NO_3_^-^-N) and ammonium nitrogen (NH_4_^+^-N) the mineral N content after 2 weeks of pre-flooding for pot experiment, total nitrogen (TN), organic matter (OM), carbon-nitrogen ratio (C/N), clay content (Clay), silt content (Silt), sand content (Sand) in Jurong (JR) and Yancheng (YC) soils.

Soil	Site	Latitude, longitude	pH	NO_3_^-^-N (mg kg^-1^)	NH_4_^+^-N (mg kg^-1^)	TN (g kg^-1^)	OM (g kg^-1^)	C/N	Clay (%) <2 um	Silt (%) 2-20 um	Sand (%) 20-2000 um
JR	Jurong	28°15´N, 116°55´E	5.94 ± 0.02	12.63 ± 0.00	24.48 ± 0.37	0.96 ± 0.01	14.30 ± 0.30	8.64 ± 0.24	35.6 ± 0.26	35.9 ± 0.28	28.5 ± 0.21
YC	Yancheng	33°43´N, 118°86´E	8.38 ± 0.02	42.25 ± 0.00	6.04 ± 0.00	1.20 ± 0.03	15.93 ± 0.11	7.70 ± 0.19	21.8 ± 0.22	58.4 ± 0.24	19.8 ± 0.09

### 2.2 Pot experiment

The greenhouse of Yangzhou University’s Yangzijin campus in Yangzhou City, Jiangsu Province was used for the potted rice experiment. Rice was cultivated using the root-bag potting method to obtain soil samples from the root and non-root zones at different stages. The cultivation pots were made of polyethylene material, 30 cm in height and 15 cm in inner diameter. The root bags had a mesh size of 300 and a diameter of 10 cm, with a height of 10 cm. Each nylon mesh bag was filled with 1 kg of air-dried soil, and 6 kg of air-dried soil was added outside the nylon bag. Calcium superphosphate was used as the phosphorus fertilizer, with a dosage of 105 kg hm^-2^ of P_2_O_5_, and potassium chloride was used as the potassium fertilizer with a dosage of 90 kg hm^-2^ of K_2_O. Both fertilizers were applied as base fertilizer at one time. Nitrogen fertilizer was applied as urea with a dosage of 220 kg hm^-2^ of N, with 60% applied as base fertilizer and 40% as top dressing. The soil and fertilizer were thoroughly mixed, and the pots and mesh bags were filled, watered evenly, and compacted.

The rice plants were cultivated using the tray-seeding method. When the seedlings grew to a height of 15 cm, they were transplanted into root bags (300 mesh nylon yarn mesh, diameter 8cm, height 15 cm) with three seedlings per net bag and three repetitions per growth stage. Regular observations were made based on the growth stage of the rice to manage water and fertilizer. Before the rice matured, the water depth was maintained at 2 - 3 cm, and soil moisture was gradually reduced after entering maturity. At each rice growth stage, three pots from the JR and YC varieties were randomly sampled. The nylon bags containing roots were carefully removed from each pot, and rhizosphere soil samples were obtained using the shaking method. Briefly, rhizosphere soil defined as the soil adhering to the root after gentle shaking. After removing all root pieces, the rhizosphere soils (labeled as R soils) were placed in sterilized plastic bags. Soil from outside the root bag was designated as non-rhizosphere soil (labeled as NR soil). Soil samples used for N_2_O indoor culture experiment and net nitrification rate were air-dried at 4°C to achieve 40% - 50% moisture content and passed through a 2 mm sieve and thoroughly blended and keep at 4°C before the incubation experiment. Soils samples used for DNA extraction, amplification were frozen at -80°C. Soil samples used for chemical analyses were aired-dried. The temperature inside the greenhouse was maintained at 30 ± 3°C during the day and 20 ± 3°C at night throughout the pot experiment. Humidity and natural light conditions were also maintained throughout the experiment.

### 2.3 Indoor culture experiment

The N_2_O flux rate and net nitrification rate of rice soils were determined following the method of Yang et al. (2019). Fresh soil samples were collected from both the rhizosphere (R) and non-rhizosphere (NR) areas at various rice growth stages. These samples were immediately used for controlled incubation experiments following these specific steps: 20 g of air-dried soil sample (by weight) was placed in a 250 ml triangular flask and adjusted the soil water content to 40% field capacity. The flask was sealed with perforated cling film and incubated in a constant temperature and humidity incubator at 25°C for 1 day. After soil stabilization, 50 mg N kg^-1^ ammonium sulfate solution was added to each triangular flask, and then soil water content adjusted to 60% field capacity, which were the optimum conditions for nitrification activities for most soils (Yang et al., 2019). The triangular flasks were sealed with stoppers containing small holes to allow gas exchange, except during gas sampling. The flasks were incubated in a constant temperature and humidity incubator at 25°C for 7 days. Every 2 days, the flasks were weighed to account for water loss due to evaporation, and the necessary amount of water was added to compensate.

N_2_O production rates were determined by measuring the accumulation of gas in the headspace over a 4-hour period on days 1, 2, 3, 5, and 7 of incubation. For each sampling day, three flasks per soil type were used, while the remaining flasks were kept under the same incubation conditions. The procedure for measuring N_2_O production was as follows: Prior to the 4-hour accumulation period, the flasks were sealed with airtight silicone rubber stoppers equipped with butyl rubber septa and 704 silicone gel to ensure an airtight seal. The flasks were connected to a multiport vacuum manifold and subjected to a vacuum, followed by flushing with fresh air, repeated three times (each for approximately 10s) to expel air from the flasks. The N_2_O concentration of the fresh air was used as the baseline concentration at the start of the incubation. The flasks were incubated at 25°C, and after 4 hours, 20 mL of headspace gas was sampled using a gas-tight syringe and stored in 20 mL pre-evacuated vials. The effective volume of the flasks was adjusted according to the water volume added. After gas collection, the soil samples in the triangular flasks were placed in sterilized bags and stored in a -80°C freezer for the determination of nitrifying microbial functional genes’ abundance and diversity.

### 2.4 Incubation with nitrification inhibitors

To explore the effects of nitrification inhibitor (NI) addition on N_2_O production in rhizosphere and non-rhizosphere soils, we conducted another indoor cultivation experiment using the methodology outlined in reference (Yang et al., 2019). We specifically selected growth stages where the cumulative N_2_O emissions in the rhizosphere soil were significantly higher than those in the non-rhizosphere soil. Additionally, these stages corresponded to the periods when the total cumulative N_2_O emissions from both rhizosphere and non-rhizosphere soils were the highest across the four rice growth stages for both JR and YC soil types. A 7-day soil incubation experiment was performed to compare the N_2_O emissions across four soil samples: JR-Rhizosphere (JR-R), JR-Non-rhizosphere (JR-NR), YC-Rhizosphere (YC-R), and YC-Non-rhizosphere (YC-NR), with the application of NI. The NI used was dicyandiamide (DCD), applied at a rate of 10% of the N added concurrently with ammonium sulfate in solution. The procedures for incubation and gas sampling for N_2_O emissions followed the same methodology as described above for the indoor cultivation experiment.

### 2.5 Analyses

The soil properties, including soil pH, ammonium nitrogen (NH_4_^+^-N), nitrate nitrogen (NO_3_^–^-N), available phosphorus (AP), available potassium (AK), total nitrogen (TN), organic matter (OM), electrical conductivity (EC), total phosphorus (TP), and soil texture, were analyzed following the procedures outlined in Soil Agro-Chemical Analyses (Lu, 2000). The net nitrification rate of the rice soil was determined using the method described by Yang et al. (2017).

Nitrous oxide (N_2_O) concentrations were measured using an Agilent 7890 gas chromatograph equipped with an electron capture detector (ECD) maintained at 300°C. The column temperature was set at 40°C, and argon-methane (5%) was used as the carrier gas at a flow rate of 30 ml min^-1^. N_2_O fluxes were calculated using the equation provided by Yang et al. (2019).

The DNA from rice soil samples at each stage was extracted using the Fast DNASPIN KIT. The purity and concentration of the DNA were assessed using NanoDrop2000, and the integrity was shown by agarose gel electrophoresis. To quantify the abundance of functional genes for ammonia-oxidizing archaea (AOA) and ammonia-oxidizing bacteria (AOB), a LightCycler ST300 (Roche Diagnostics, Germany) real-time PCR instrument was used. The primers Arch-amoAF/Arch-amoAR and amoA1/FamoA2R were used for AOA and AOB amplification, respectively, and the instructions of the kit were followed for the specific operations. The diversity of AOA and AOB was determined by Shanghai Majorbio Bio-pharm Technology Co., Ltd.

### 2.6 Calculation and statistical analyses

The N_2_O flux rates, expressed as µg N kg^-1^ soil h^-1^, were calculated based on the rate of increase in N_2_O concentrations in the headspace of the incubation flasks. To enable better comparison across treatments, the N_2_O emission fluxes were presented as time-weighted averages, representing the average N_2_O emissions. Cumulative N_2_O emissions were determined by summing the products of the average emission rates between two consecutive measurements and the time intervals between those measurements, following the method described by Zhou et al. (2019), as shown in (Equation 1):


(1) $$E=\sum \frac{\left(f_{i}+f_{i+1}\right)}{2}\times (t_{i+1}-t_{i})$$

where *E* represents the cumulative N_2_O emissions; *t*_i+1_-*t*_i_ is time interval between two consecutive measurements; and *f*_i_ and *f*_i+1_ are the emission rates of N_2_O at time *t*_i_ and *t*_i+1_, respectively. All data shown are the means of three replicates and statically analyzed by General Linear Model (GLM-ANOVE) in SPSS 16.0.

The Student’s *t*-test was used to assess differences in N_2_O flux, N_2_O cumulative emissions, AOA and AOB *amoA* gene copy numbers, and diversity between rhizosphere and non-rhizosphere soils at the same rice growth stage within the same paddy soil. It was also applied to compare differences between JR and YC soils in both rhizosphere and non-rhizosphere soils at the same growth stage. ANOVA followed by LSD’s test was used to identify significant differences in N_2_O flux, N_2_O cumulative emissions, *amoA* gene copy numbers, and diversity of AOA and AOB among the four rice growth stages. Spearman’s rank correlation analysis was performed to explore the relationship between N_2_O cumulative emissions and *amoA* gene copy numbers, as well as the diversity of nitrifying microorganisms. Principal component Analysis (PCA) was employed to assess the impact of soil properties on N_2_O cumulative emissions. Additionally, Redundancy Analysis (RDA) was used to investigate the combined effects of soil properties and nitrifying microorganisms on N_2_O accumulation emissions.

## 3 Results

### 3.1 Difference in N_2_O flux between rhizosphere and non-rhizosphere paddy soil

**Figure 1:**
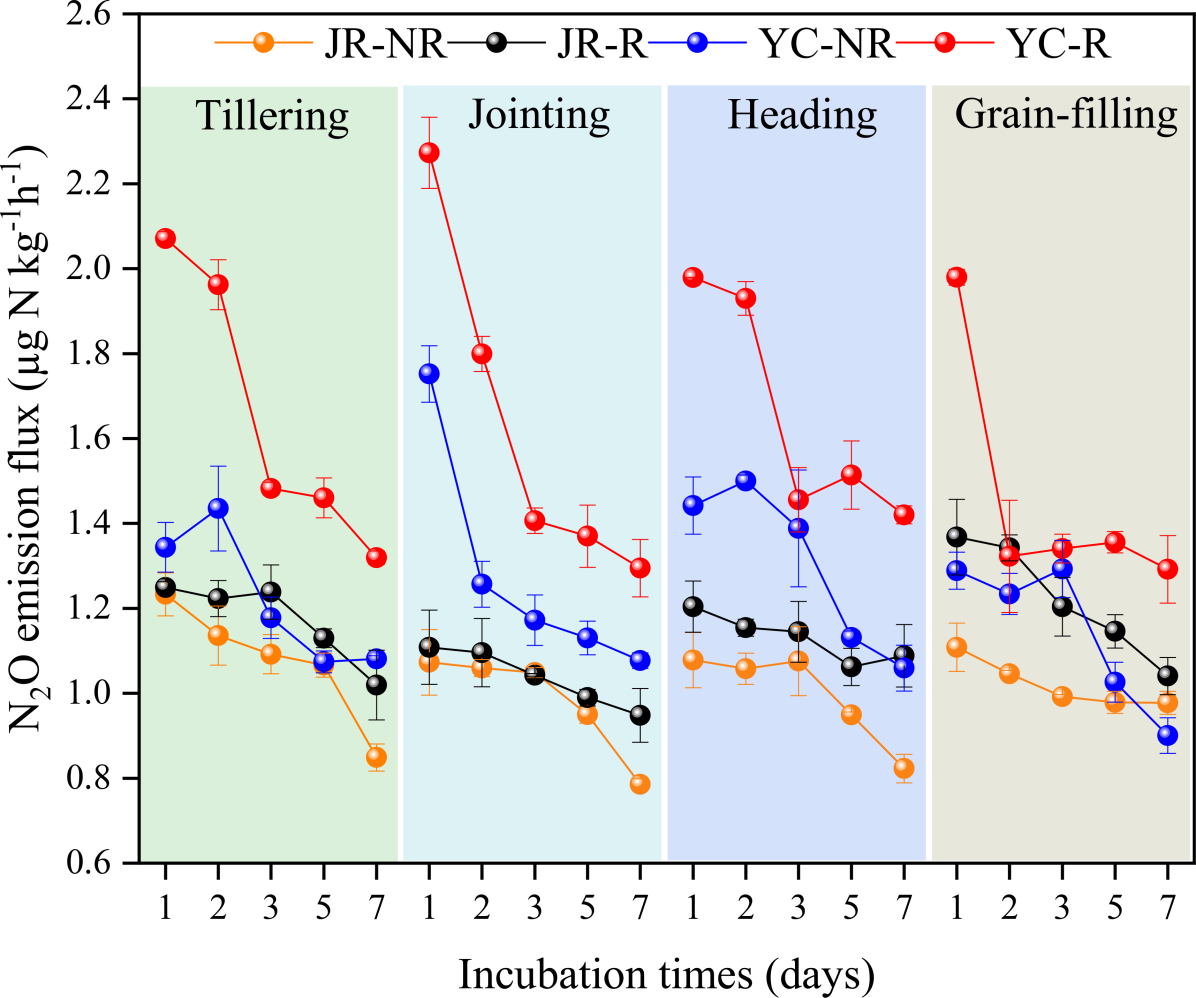
N_2_O flux from rhizosphere (R) and non-rhizosphere (NR) in Jurong (JR) and Yancheng (YC) at rice tillering, jointing, heading and grain-filling stages during the incubation at 25°C. Bars are standard deviation of the means.

Rhizosphere soil was the predominant source of N_2_O production during the entire growth cycle of the rice. The N_2_O flux ranged from 0.95-1.37 µg N kg^-1^ h^-1^ and 1.29-2.27 µg N kg^-1^ h^-1^ in JR-R and YC-R, respectively, while it ranged from 0.79-1.23 µg N kg^-1^ h^-1^ and 0.90-1.75 µg N kg^-1^ h^-1^ in JR-NR and YC-NR (**Figure 1**). The average N_2_O flux in rhizosphere paddy soils was significantly higher than that from non-rhizosphere paddy soils (*P<* 0.05). Similarly, cumulative N_2_O emissions from JR-R and YC-R soils were significantly greater than those from JR-NR and YC-NR soils across the entire growth period (*P*< 0.05), accounting for 51.19%-54.55% and 55.74%-57.62% of the total N_2_O emissions from JR and YC soils, respectively (**Table 2**).

**Table 2:** N_2_O cumulative emissions and contributions of rhizosphere and non-rhizosphere soils in Jurong (JR) and Yancheng (YC) during a 7-day incubation.

Soil	Stage	N_2_O emission (µg kg^-1^)			Relative contribution (%)		
		Non-rhizosphere	Rhizosphere	Total*	Non-rhizosphere	Rhizosphere	Stage*
JR	Tillering	183.25 ± 2.66 bB(a)	198.63 ± 0.39 aB(ab)	381.88 ± 2.53B(a)	47.99	52.01	26.31
	Jointing	165.92 ± 3.24 bB(c)	174.03 ± 4.52 aB(c)	339.95± 6.79B(c)	48.81	51.19	23.42
	Heading	168.41 ± 1.84 bB(bc)	184.99 ± 1.95 aB(b)	353.40± 1.95B(b)	47.65	52.35	24.35
	Grain-filling	170.98 ± 0.57 bB(b)	205.25 ± 3.73 aB(a)	376.23± 7.30B(a)	45.45	54.55	25.92
Total		688.56 ± 4.10 bB	762.90 ± 5.03 aB	1451.46 ± 48.46B	47.44	52.56	
YC	Tillering	202.52 ± 4.92 bA(bc)	275.39 ± 7.19 aA(a)	477.90 ± 17.92A(a)	42.38	57.62	25.11
	Jointing	215.35 ± 6.02 bA(ab)	271.19 ± 4.35 aA(a)	486.54 ± 14.92A(a)	44.26	55.74	25.56
	Heading	219.12 ± 7.56 bA(a)	276.72 ± 8.98 aA(a)	495.84 ± 6.88A(a)	44.19	55.81	26.05
	Grain-filling	194.21 ± 0.65 bA(c)	249.03 ± 2.18 aA(b)	443.24 ± 12.81A(b)	43.82	56.18	23.29
Total		831.20 ± 3.64 bA	1072.32 ± 10.14 aA	1903.52± 437.82A	43.67	56.33	

Different lowercase letters (a, b) following values indicate significant differences between rhizosphere and non-rhizosphere in the same soil and same rice stage at *P<* 0.05 (*t*-test). Different uppercase letters (A, B) indicate significant differences between JR and YC in the same non-rhizosphere, same rhizosphere soil and same rice stage at *P<* 0.05 (*t*-test). Different lowercase letters in brackets (a, b, c) indicate significant differences among different rice stages in the same non-rhizosphere soil, same rhizosphere soil at *P<* 0.05 (LSD-test). “Total” represents the cumulative N_2_O emissions from the same non-rhizosphere and rhizosphere soils across all rice growth stages. “Total*” denotes the sum of N_2_O emissions from both non-rhizosphere and rhizosphere soils within the same rice stage. “Stage*” refers to the contribution of a specific growth stage (Tillering, Jointing, Heading, or Grain-filling) to the overall N_2_O emissions from either non-rhizosphere or rhizosphere soils.

### 3.2 Differences in N_2_O flux among various growth stages

Significant differences in cumulative N_2_O emissions were observed among the various growth stages within the same rice soil (*P<* 0.05) (**Table 2**). Specifically, the N_2_O cumulative emission during the grain-filling stage was significantly lower than in the other three rice stages for both YC-NR and YC-R soil. Similarly, during the jointing stage, the N_2_O cumulative emission was significantly lower than in the tillering and grain-filling stages for both JR-NR and JR-R soil. The proportions of total N_2_O emissions attributed to the tillering, jointing, heading, and grain-filling stages were 26.31%, 23.42%, 24.35%, and 25.92% in JR soil, respectively, and 25.11%, 25.56%, 26.05%, and 23.29% in YC soil, respectively. Summing up the N_2_O cumulative emissions from R and NR soils at the same growth stage, the total N_2_O cumulative emissions from YC soil were significantly higher than those from JR soil (*P<* 0.05).

### 3.3 Changes in nitrifying community

**Figure 2:**
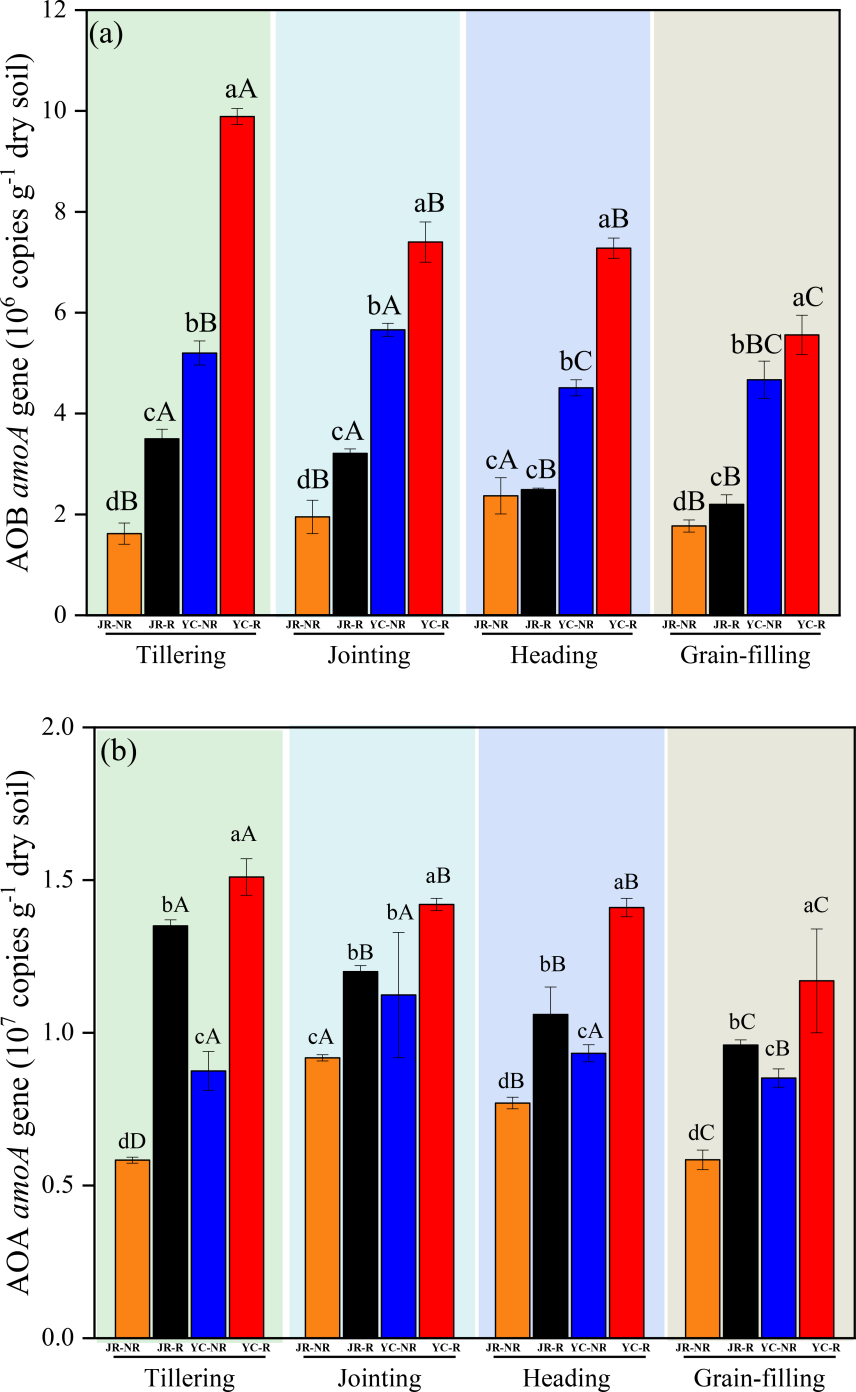
Variations in AOB **(A)** and AOA **(B)** *amoA* gene number in Jurong (JR) and Yancheng (YC) rhizosphere (R) and non-rhizosphere (NR) soils with four rice stages (Tillering, Jointing, Heading, and Grain-filling). Bars are standard deviation of the means. Different lowercase letters (a–d) following values indicate significant differences among JR-R, JR-NR, YC-R and YC-NR in the same rice stage at *P<* 0.05 (LSD-test). Different uppercase letters (A–C) indicate significant differences among four rice stages in the same soil at *P<* 0.05 (LSD-test).

The *amoA* gene copy numbers of AOB and AOA were significantly higher in JR-R and YC-R soils compared to JR-NR and YC-NR soils throughout the rice growth period, except for the AOB copy numbers at the heading stage in JR soil (*P*< 0.05) (**Figure 2A**). Significant differences in amoA gene copy numbers were also observed among different growth stages (*P*< 0.05). Specifically, the copy numbers of both AOA and AOB were significantly lower during the grain-filling stage compared to the other three stages in both JR and YC soils (**Figure 2**).

**Figure 3:**
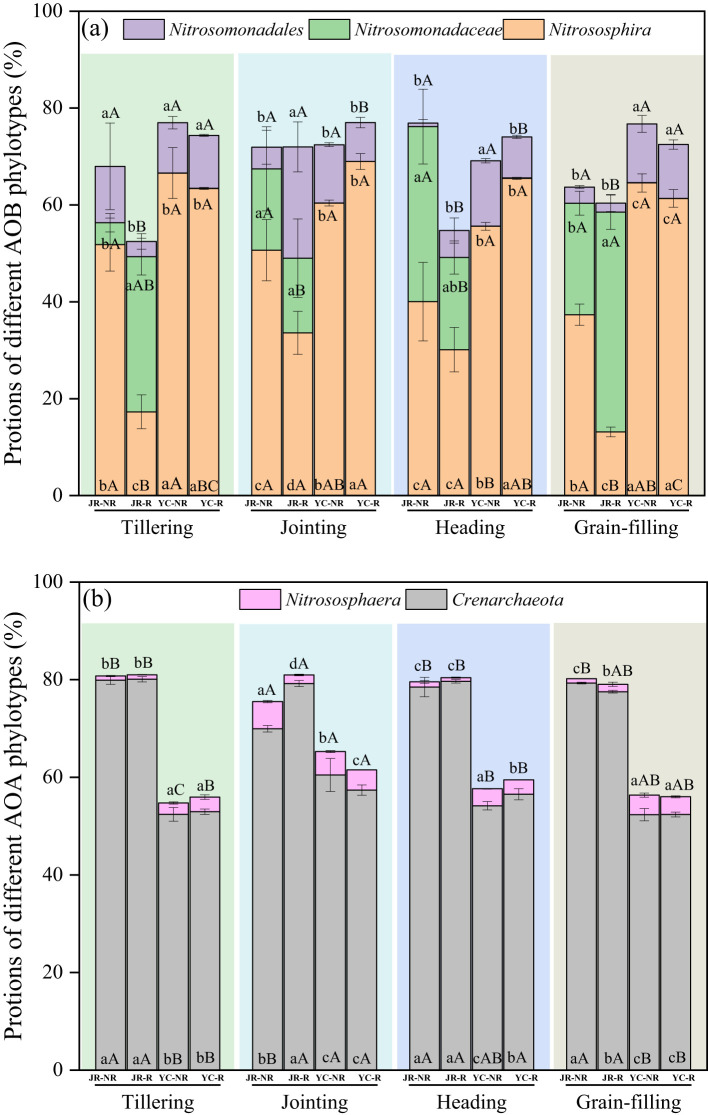
Relative abundance of AOB **(A)** and AOA **(B)** at the phylotypes level in JR (Jurong) and YC (Yancheng) rhizosphere (R) and non-rhizosphere (NR) soils with four rice stages (Tillering, Jointing, Heading, and Grain-filling). Bars are standard deviation of the means. Different lowercase letters (a–d) following values indicate significant differences among JR-R, JR-NR, YC-R and YC-NR in the same rice stage at *P<* 0.05 (LSD-test). Different uppercase letters (A–C) indicate significant differences among four rice stages in the same soil at *P<* 0.05 (LSD-test).

The diversity of AOB exhibited substantial variation between R and NR soils, as well as among the four rice growth stages in both JR and YC soils (**Figure 3A**). *Nitrosospira* was the dominant AOB genus in YC paddy soils across all growth stages, accounting for 52.34%-60.47% of the AOB community, which was significantly higher than its proportion in JR soils. In contrast to AOB, the diversity of AOA showed minimal variation between R and NR soils, as well as among the four rice growth stages in both JR and YC soils (**Figure 3A**). *Crenarchaeota* was the dominant AOA phylum in both JR and YC soils across all stages, and its relative abundance was significantly higher in JR soils compared to YC soils (**Figure 3B**).

### 3.4 Factors affecting N_2_O emissions

**Figure 4:**
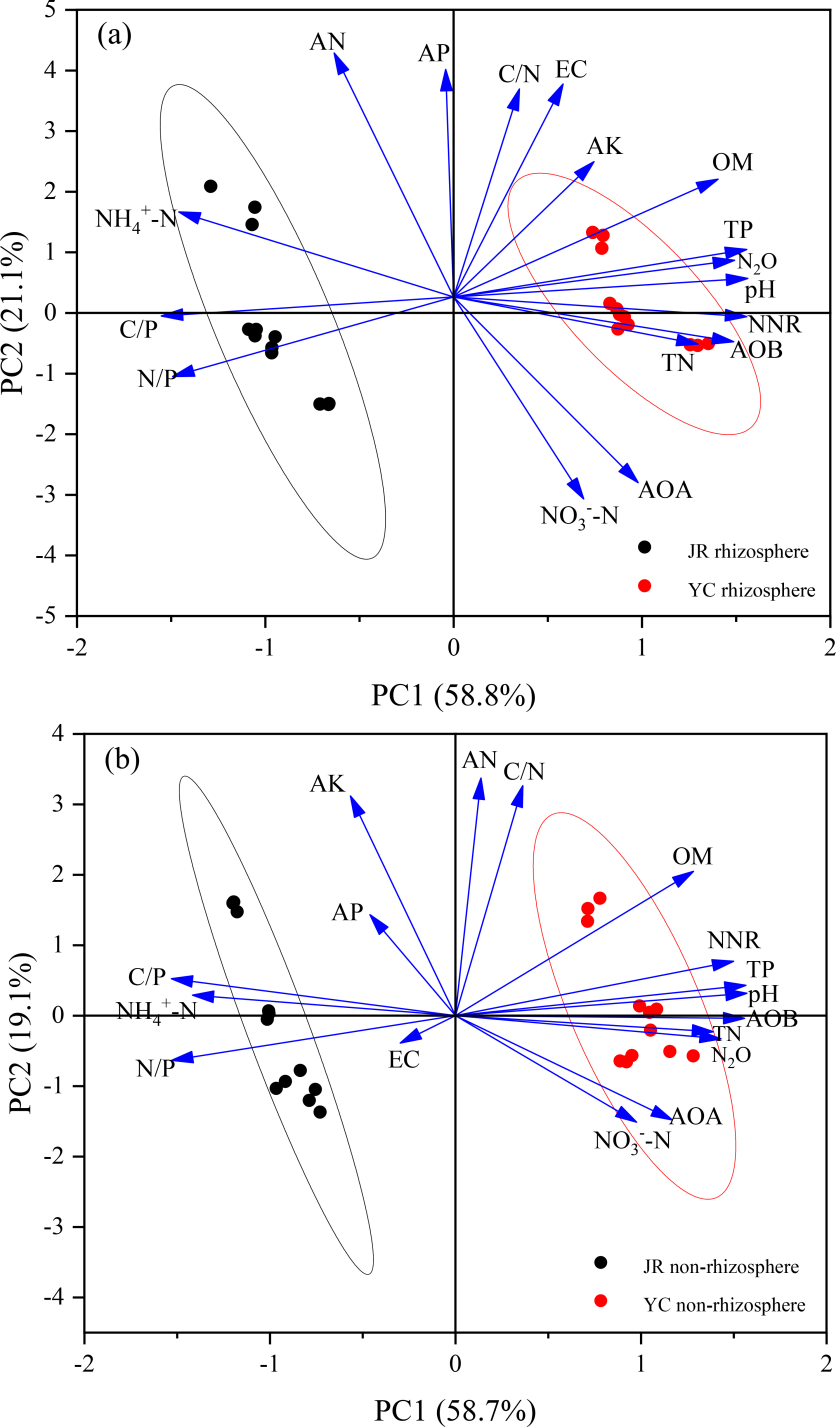
Scores and loadings of the principal component analysis (PCA) revealing the impact of soil properties on N_2_O accumulation emissions in rhizosphere **(A)** and non-rhizosphere **(B)** soils. EC, electrical conductivity; AP, available phosphorus; AK, available potassium; AN, alkaline nitrogen; TN, soil total nitrogen; TP, total phosphorus; OM, organic matter; pH, pounds Hydrogen; NH_4_^+^-N, ammonium nitrogen; NO_3_^-^-N, nitrate nitrogen; C/N, organic carbon to total nitrogen ratio; C/P, organic carbon to total phosphorus ratio; N/P, total nitrogen to total phosphorus ratio; NNR, net nitrification rate; AOB, gene copy number of AOB; AOA, gene copy number of AOA; N_2_O, N_2_O cumulative emissions.

Soil characteristics were found to be the primary determinants of N_2_O emissions, as evidenced by the clear differentiation along the PC1 axis between JR-R and YC-R soils. This differentiation is further supported by the fact that Principal component 1 (PC1) accounted for 58.8% of the variance in N_2_O emissions from these soils, as depicted in **Figure 4A**. The variables with positive loadings on PC1 include pH, total phosphorus (TP), organic matter (OM), available potassium (AK), electrical conductivity (EC), carbon to nitrogen ratio (C/N), net nitrification rate (NNR), ammonia-oxidizing bacteria (AOB), total nitrogen (TN), ammonia-oxidizing archaea (AOA), and nitrate nitrogen (NO_3_^-^-N). Conversely, negative loadings were observed for ammonium nitrogen (NH_4_^+^-N), carbon to phosphorus ratio (C/P), and nitrogen to phosphorus ratio (N/P), which were identified as critical factors affecting N_2_O emissions specifically in JR-R soil. In addition to PC1, Principal component 2 (PC2) explained 21.1% of the variance and showed a positive association with soil available nitrogen (AN) and available phosphorus (AP).

**Figure 5:**
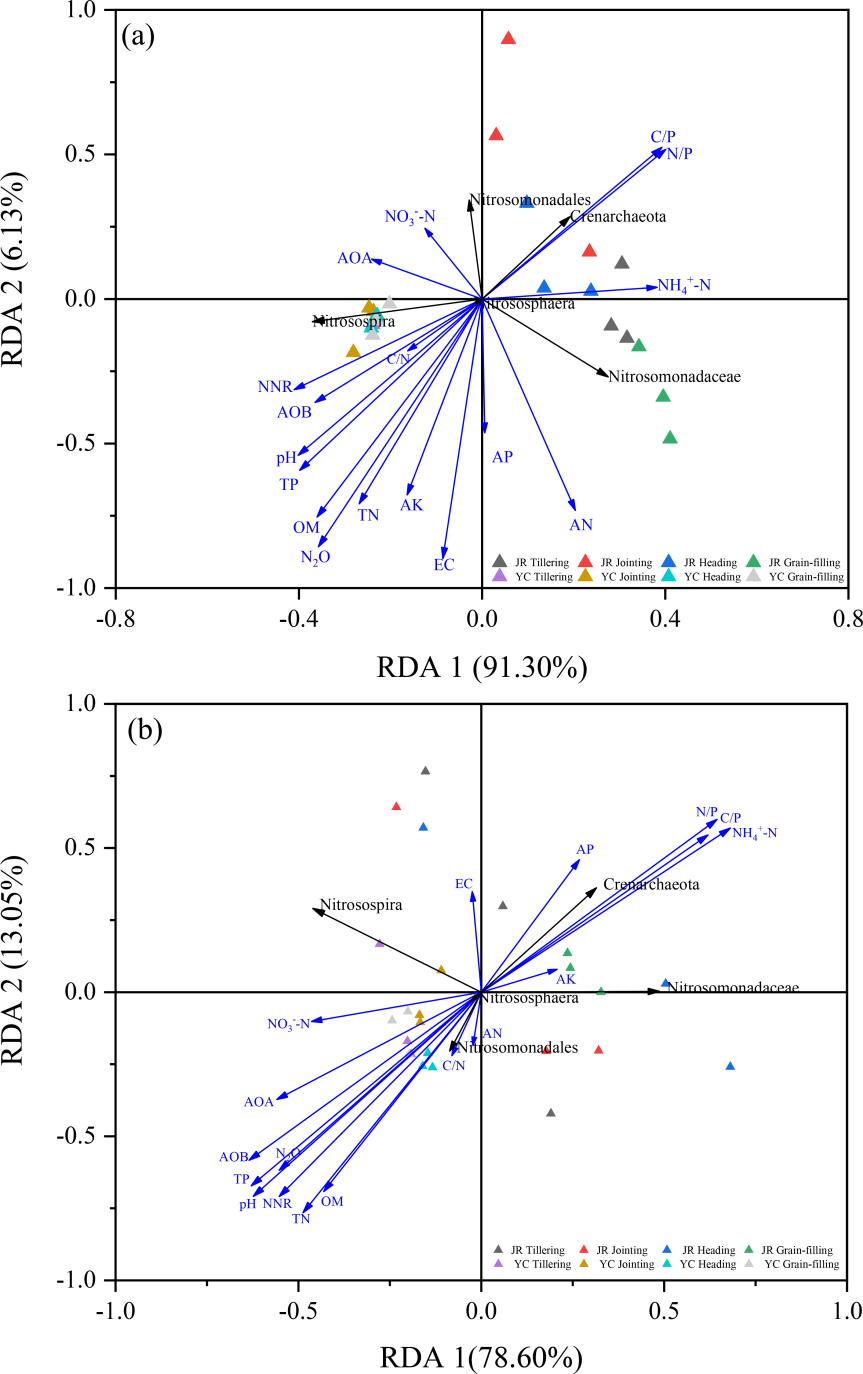
Redundancy analysis (RDA) showing the effects of soil properties and relative abundance of nitrifying microorganism on N_2_O accumulation emissions in rhizosphere soils **(A)** and non-rhizosphere soils **(B)**. EC, electrical conductivity; AP, available phosphorus; AK, available potassium; AN, alkaline nitrogen; TN, soil total nitrogen; TP, total phosphorus; OM, organic matter; pH, pounds Hydrogen; NH_4_^+^-N, ammonium nitrogen; NO_3_^–^-N, nitrate nitrogen; C/N, organic carbon to total nitrogen ratio; C/P, organic carbon to total phosphorus ratio; N/P, total nitrogen to total phosphorus ratio; NNR, net nitrification rate; AOB, gene copy number of ammonia-oxidizing bacteria; AOA, gene copy number of ammonia-oxidizing archaea gene; N_2_O, nitrous oxide cumulative emissions.

The separation of points along the PC1 axis between JR-NR and YC-NR soils indicated that N_2_O emissions were predominantly influenced by soil differences, highlighting distinct emission profiles in each soil type. Principal component 1 (PC1) explained 58.7% of the variance in N_2_O accumulative emissions in JR-NR and YC-NR soils, as shown in **Figure 5B**. Positive loadings were associated with TN, AOB, pH, TP, NNR, OM, AOA, and NO_3_^-^-N. In contrast, strong negative loadings were observed for EC, N/P, NH_4_^+^-N, and C/P. Principal component 2 (PC2) explained 19.1% of the variance, with positive associations for AK and AP.

We employed redundancy analysis (RDA) to delve deeper into the factors influencing the distribution of AOA and AOB phyla in JR-R and YC-R soils, as depicted in **Figure 5A**, and in JR-NR and YC-NR soils, shown in **Figure 5B**. For JR-R and YC-R soils, RDA 1 and RDA 2 explained 91.3% and 6.13% of the variation in N_2_O accumulation emissions, respectively. The C/N, pH, TP, and OM were identified as the primary factors influencing the *Nitrosospira* genus of AOB. C/N and carbon to phosphorus ratio (C/P) were the main influences on the *Crenarchaeota* phylum of AOA. In the case of JR-NR and YC-NR soils, RDA 1 accounted for 78.6% of the variance, while RDA 2 explained 13.05%. Notably different from the results in R soils, in NR soils, the *Nitrosomonadales* order was predominantly affected by factors such as C/N, OM, and TN, indicating a distinct influence of soil characteristics on different microbial communities responsible for N_2_O emissions.

### 3.5 Effect of DCD on N_2_O emissions

**Figure 6:**
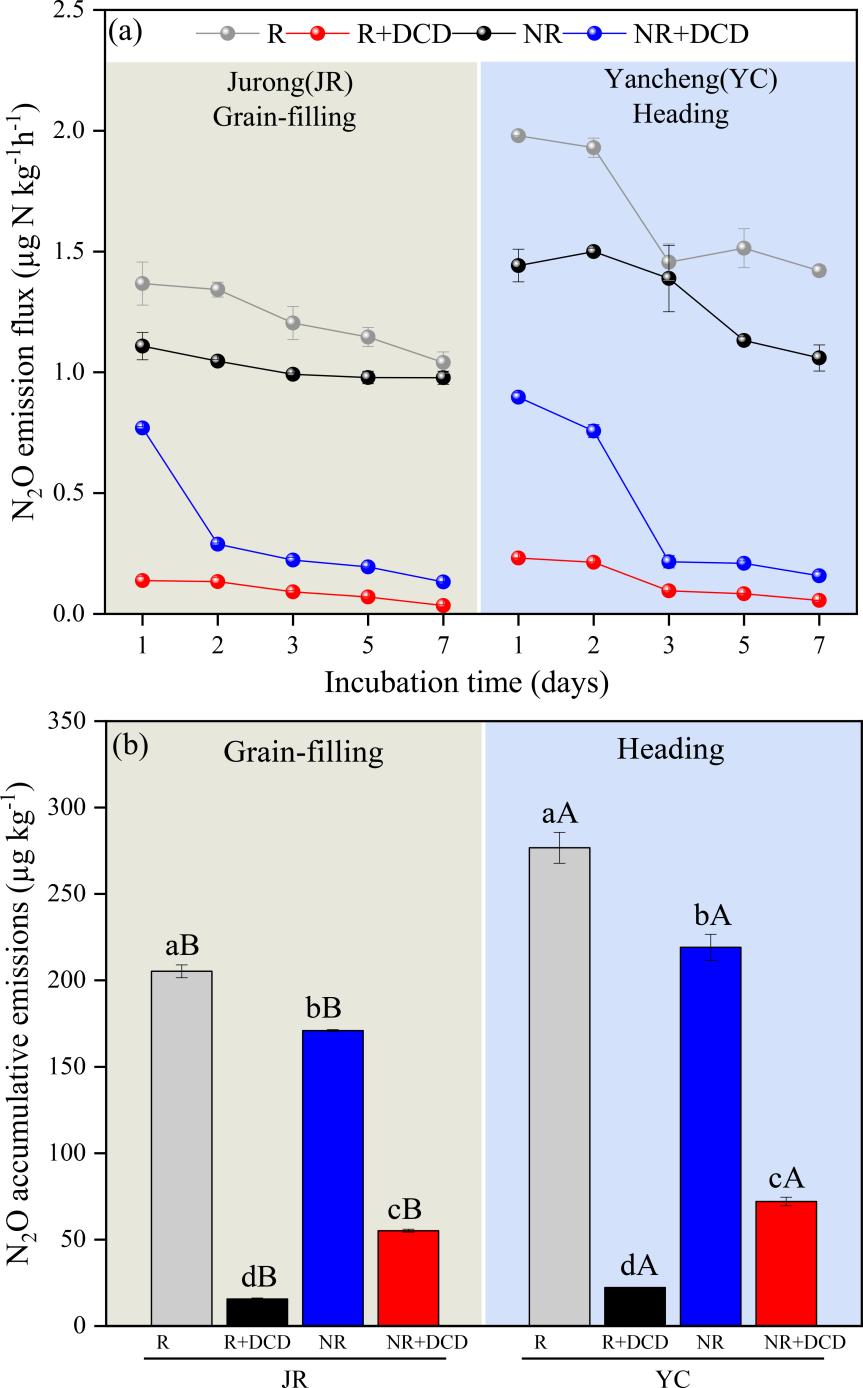
Comparison of the effect of added nitrification inhibition (DCD) on N_2_O flux **(A)** and accumulation emissions **(B)** by soils in Jurong rhizosphere (JR-R) and non-rhizosphere (JR-NR), Yancheng rhizosphere (YC-R) and non-rhizosphere (YC-NR) incubated at 25°C. Bars are standard deviation of the means. Different lowercase letters (a, b) indicate significant differences between JR-R and JR-NR, YC-R and YC-NR (*t*-test); Different uppercase letters (A, B) indicate significant differences between JR-R and YC-R, JR-NR and YC-NR (*t*-test).

The addition of NIs significantly influenced the N_2_O fluxes and cumulative emissions in both JR-R, YC-R and JR-NR, YC-NR soils (**Figures 6A, B**). For the rhizosphere soils (JR-R and YC-R), the N_2_O fluxes ranged from 0.034 to 0.138 µg kg^-1^ h^-1^ for JR-R and 0.056 to 0.231 µg kg^-1^ h^-1^ for YC-R. The use of NIs at the grain-filling stage led to a dramatic reduction in cumulative N_2_O emissions, showing a decrease of 92.37% for JR-R and 91.93% at the tillering stage for YC-R. In the non-rhizosphere soils (JR-NR and YC-NR), the emission fluxes were notably higher, ranging from 0.133 to 0.770 µg kg^-1^ h^-1^ for JR-NR and 0.157 to 0.897 µg kg^-1^ h^-1^ for YC-NR. Here, the impact of NIs was also significant but less pronounced compared to the R soils, reducing cumulative emissions by 67.73% at the grain-filling stage for JR-NR and by 64.42% at the tillering stage for YC-NR.

## 4 Discussion

### 4.1 The influence of rhizosphere effects on N_2_O emissions

The study results revealed that N_2_O production from rice R soil were significantly higher than those from NR soil during key growth stages of rice (tillering, jointing, heading, and grain-filling) in both JR and YC soils (**Table 2**), which is consistent with previous research (Nie et al., 2014). The phenomenon can be attributed to several factors. Firstly, rice rhizosphere soil contains a higher carbon content due to the presence of rice root exudates, which are low-molecular-weight carbon compounds such as sugars, organic acids, and amino acids that are easily decomposed and utilized by microorganisms. This, in turn, promotes the activity of nitrifying microorganisms and N_2_O emissions (Liyanage et al., 2022; Kong et al., 2021; Liu et al., 2021). Secondly, although the pH of R soil is lower than that of NR soil (**Supplementary Table S1**), the lower pH did not suppress N_2_O emissions from YC-R and JR-R soil. This is possibly because AOA has a high affinity for NH_3_ under relatively low pH conditions, promoting both nitrification and N_2_O emissions (Hu et al., 2020; Azziz et al., 2016; Liu et al., 2017).

**Figure 7:**
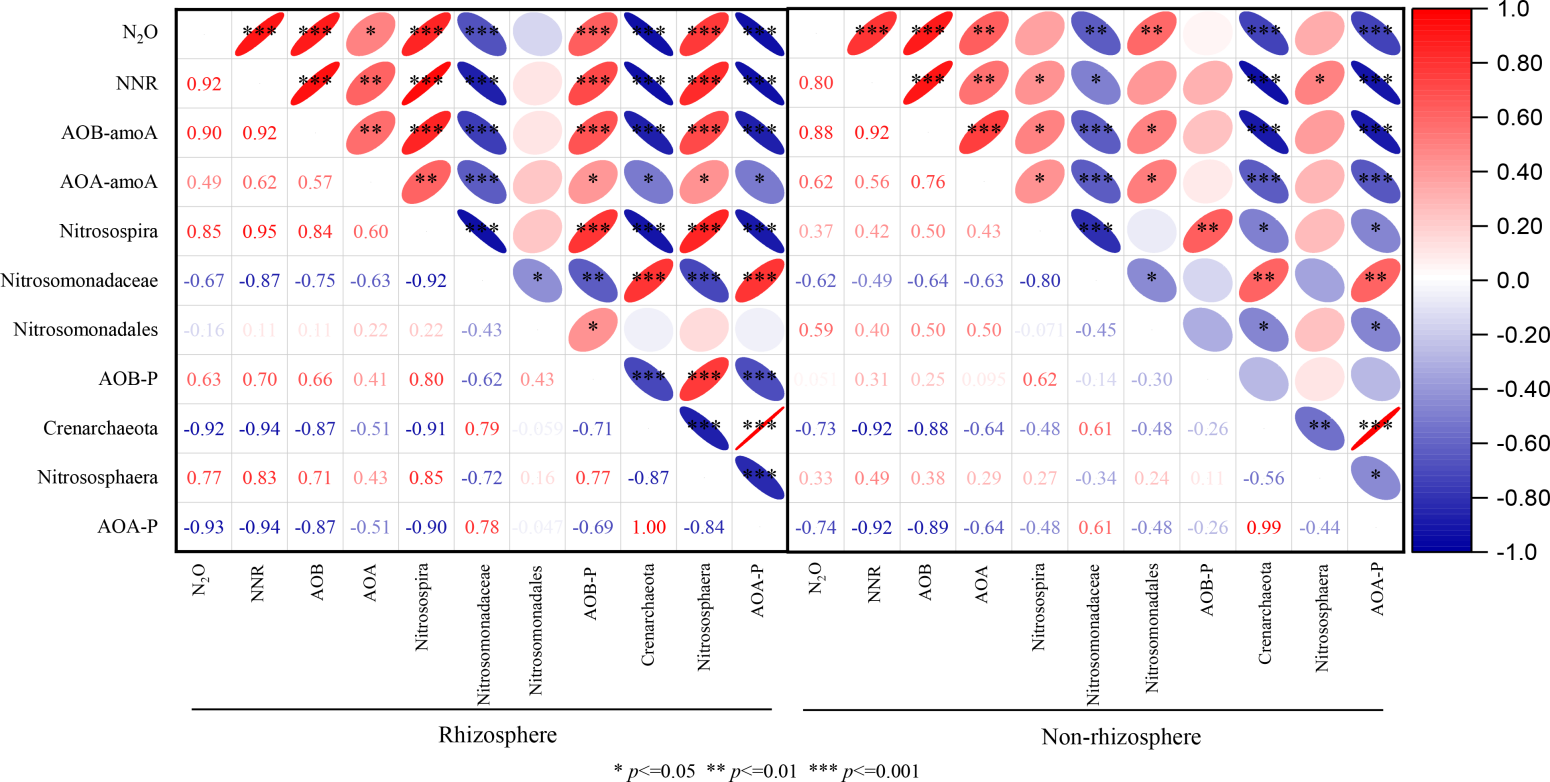
Spearman’s rank correlation coefficient between N_2_O accumulation emissions and nitrification rate, *amoA* gene copies numbers, diversity of nitrifying microorganisms in rhizosphere **(A)** and non-rhizosphere soils **(B)** (*n* = 24, two-tailed). N_2_O, N_2_O accumulation emissions; NNR, net nitrification rate; AOB, *amoA* gene number of AOB; AOA, *amoA* gene number of AOA; AOB-P, total relative abundance of *Nitrosospira*, *Nitrosomonadales* and *Nitrosomonadales*; AOA-P, total relative abundance of *Crenarchaeota* and *Nitrosomonadales*.

In this study, the relative abundance of AOA and AOB in rhizosphere and non-rhizosphere were different and consistent with previous studies (Dong et al., 2021; Angel et al., 2010), their results have shown that rice root exudates and rice root absorb nitrogen directly changed R soil physical and chemical properties then changes nitrification rates, indirectly affecting composition of the nitrification microbial community. *Nitrosospira* oxidizes ammonia to nitrous acid showed positive correlations with N_2_O accumulation emissions in R soils (**Figure 4**). *Nitrosospharea* is involved nitrite oxidation process also showed positive correlations in R soils with N_2_O production (**Figure 7**). Therefore, we concluded that N_2_O emission was influenced by R soil characters and also the community and abundance nitrification microbiomes.

The use of nitrification inhibitors (NIs) such as dicyandiamide (DCD) significantly diminished N_2_O accumulation emissions in both JR-R and YC-R soils, demonstrating reductions between 92.37% and 93.93% as compared to JR-NR and YC-NR. This range of reduction aligns with previously reported results in the literature, where reductions span from 0% to 96% (Lyu et al., 2021; Lu et al., 2024). The variability in these figures reflects the differential effectiveness of NIs, primarily influenced by their ability to slow down the process of ammonia oxidation. The notably better performance of DCD in reducing N_2_O emissions in R soils as opposed to NR soils can likely be attributed to the distinctive physical and chemical properties of these soil types, as well as the differing communities of nitrifying microorganisms present. Rhizosphere soils generally have enhanced microbial activity and different nutrient dynamics due to the proximity to plant roots, which can alter the effectiveness of NIs. Such differences underscore the importance of considering soil-specific characteristics and microbial populations when evaluating the efficacy of nitrification inhibitors in agricultural settings.

### 4.2 The impact of rice growth stages on N_2_O emissions

Rice growth stages play a critical role in influencing N_2_O production and emissions from paddy soils (Xu et al., 2022a; Kim et al., 2021). Variations in N_2_O emissions across different rice growth stages are often attributed to changes in rice root activity, including oxygen diffusion through aerenchyma to the rhizosphere. This diffusion affects the stimulation of N_2_O emissions through nitrification and denitrification processes (Zhang et al., 2017; Yang et al., 2021). Xu et al. (2022a) reported that during the vigorous growth stages of rice, oxygen secretion by roots facilitates nitrification-derived N_2_O production, which is regulated by rhizosphere microorganisms. Additionally, rice root exudate composition and content vary with growth stages. He et al. (2022) demonstrated in a pot study that rice roots can release biological nitrification inhibitors to reduce nitrification rates, aligning nitrogen availability with the needs of rice plants and microorganisms. Moreover, rice roots may stimulate organic nitrogen mineralization to increase the supply of mineral nitrogen.

In this study, significant variations in N_2_O emissions were observed during the four major rice growth stages in JR and YC soils (**Table 2**). The trends in N_2_O emissions differed between the two soil types: in YC soil, lower emissions were recorded during the grain-filling stage compared to the tillering, jointing, and heading stages, whereas in JR soil, lower N_2_O emissions were observed during the jointing and heading stages compared to the tillering and grain-filling stages. It might be expected that similar trends in N_2_O emissions would occur under the same rice varieties and management practices. However, significant differences were observed in the impact of rice growth stages on JR and YC soils. These differences can be attributed to variations in soil properties (**Supplementary Table S1**) and the nitrifying microbial community (**Figures 2**, **3**) across the different growth stages in both YC and JR soils. These findings suggest that interactions between rice growth stages, soil properties, and the microbial community collectively influence N_2_O emissions. Consequently, particular attention should be given to different rice growth stages, especially within rhizosphere soils, when evaluating N_2_O emissions in paddy fields and investigating the underlying mechanisms.

### 4.3 The impact of soil properties on N_2_O emissions

Soil properties play a fundamental role in shaping the abundance and composition of AOB and AOA, and their sensitivity to these properties varies significantly (Chen et al., 2010). In this study, the diversity of AOB was more variable than that of AOA in rhizosphere soils, following a similar trend across rice growth stages (**Figures 2**, **3**). Results from PCA (**Figure 4**) and RDA (**Figure 5**) analyses indicate that AOB abundance and diversity are more strongly influenced by soil properties such as pH, TP, TN, OM, AK), EC, and C/N ratio compared to AOA. These findings suggest that rice root growth and the type and amount of root exudates significantly impact AOB abundance and diversity, ultimately affecting N_2_O production and emissions. AOB may be better adapted to changes in soil properties in rice rhizosphere soils and across different growth stages, such as lower pH, lower EC, and higher OM compared to non-rhizosphere soils (**Supplementary Table S1**). In contrast, AOA are often associated with extreme environments (Erguder et al., 2009). Further research is needed to investigate the specific types of root exudates and their effects on AOB abundance and diversity.

Soil pH, in particular, is a key factor influencing N_2_O production during both nitrification and denitrification processes (Shaaban et al., 2023). The ratio of N_2_O to NO_3_^-^ production through nitrification is often attributed to the nitrification rate, which is closely linked to soil pH. For example, when soil pH is 5.1 and 6.7, nitrification rates increase by 47% and 80%, respectively, and the N_2_O to NO_3_^-^ production ratio also rises by 36% and 23% (Cai et al., 2012). These findings highlight the critical role of soil pH in determining the extent of N_2_O emissions in paddy soils. In our study, N_2_O flux rate and cumulative emission in alkaline YC-R and YC-NR soil were higher than that in acidity JR-R and JR-NR soil, which consistent with previous research (Shaaban et al., 2023). This is mainly because soil pH significantly influences the NH_3_ substrate efficiency of nitrification, for every unit increase in pH, the concentration of NH_3_ increases by ten times. The optimal pH range for nitrifying bacteria is between 7.5 and 8.0, and nitrification generally increases with increasing soil pH. In the northern region of China, nitrification is the dominant process of N_2_O production in calcareous soils (Bozal-Leorri et al., 2021; Zhang et al., 2020). The average N_2_O flux rate ranged between 0.95 and 1.60 µg kg^-1^ h^-1^, which was lower than the results reported by Peng et al. (2024). This discrepancy may be attributed to differences in soil texture between their study and our paddy soils, as soil texture influences soil permeability, which in turn affects the rates of nitrification and denitrification. Nitrification is an aerobic process, and under well-ventilated conditions, nitrification is enhanced, leading to higher N_2_O emissions from the soil.

Several studies have shown that N_2_O emissions increase with increasing soil TN, TP and OM content (Kim et al., 2023; Lu et al., 2022). The YC soil has significantly higher TN and OM content than the JR soil (**Table 1**; **Supplementary Table S1**). This observation may account for the notable difference in N_2_O emissions between alkaline YC rice soil and acidic JR rice soil under identical cultivation conditions in our study. Additionally, the positive correlation between the abundance of AOA and AOB and soil total nitrogen (**Figure 4**) suggests that total nitrogen provides a sufficient substrate, thereby stimulating N_2_O emissions through the nitrification process.

Soil available nitrogen, encompassing NH_4_^+^-N and NO_3_^-^-N, stands out as a pivotal factor influencing N_2_O emissions (Meijide et al., 2007; Hu et al., 2013; Shaaban et al., 2019). NH_4_^+^-N, in particular, emerges as a key constituent for nitrifying microorganisms, contributing to enhanced N_2_O emissions during the rice-growth season (Xu et al., 2022b; Zhou et al., 2017). In our study, the increased N_2_O emissions were closely associated with soil available nitrogen, especially in the JR-NR and YC-NR soil (**Figure 4B**). There were notable distinctions between R and NR, with the correlation index being higher in the NR. This observation aligns with our finding that N_2_O production in our study was primarily attributed to nitrification, a process dependent on NH_4_^+^-N.

### 4.4 Implications of NIs effect on rhizosphere N_2_O emissions

Mitigation strategies for N_2_O emissions in rice paddy ecosystems primarily include water management, fertilizer management, agronomic measures, and the application of nitrification inhibitors (NIs). Among these, water management is the most critical factor influencing N_2_O emissions. Continuous flooding during the rice growing season has been shown to minimize N_2_O emissions, whereas emissions increase with the frequency of drainage events. For example, Zou et al. (2007) reported that the N_2_O emission coefficients for continuously flooded, drying-then-irrigating, and water-saving irrigation treatments were 0.02%, 0.42%, and 0.73%, respectively.

Fertilizer management is the second most influential factor affecting N_2_O emissions in paddy ecosystems. Studies have demonstrated that the type and rate of nitrogen (N) fertilizer applied significantly impact N_2_O emissions. For instance, compared with ammonium bicarbonate and urea, long-acting ammonium bicarbonate can reduce N_2_O emissions by 76%, while slow-release urea can lower emissions by approximately 58% (Li et al., 2005). However, Yan et al. (2000a) noted that slow-release fertilizers maintain high concentrations of NH_4_^+^-N during the rice drying phase, which promotes nitrification and subsequent N_2_O emissions. Additionally, when rice fields are reflooded, accumulated NO_3_^-^ is subjected to denitrification, leading to N_2_O emissions. Although slow-release fertilizers can mitigate N_2_O emissions to some extent, they may still contribute to emissions over the entire growing season and are often cost-prohibitive for widespread use. In China, over 97% of chemical N fertilizers are ammonium-based fertilizers, such as urea and ammonium bicarbonate. NIs present a promising strategy for reducing N_2_O emissions in paddy fields. Among NIs, dicyandiamide (DCD) has been widely studied and shown to reduce N_2_O emissions by 10%-60% when mixed with urea in rice field ecosystems. DCD also has the added benefit of slightly reducing CH_4_ emissions (Xu et al., 2002).

In traditional rice farming practices, nitrogen fertilizer is applied three times during the growing season: as a base fertilizer, tillering fertilizer, and ear fertilizer. Conventionally, NIs are mixed with base fertilizer and applied before rice transplanting or sowing. However, under intermittent irrigation—a common water management practice—paddy fields are continuously flooded during the period from rice transplanting to mid-season drainage, which strongly inhibits soil nitrification. As a result, NIs mixed with the base fertilizer are rendered largely ineffective. Our findings revealed that the application of NIs at the heading or grain-filling stages in rhizosphere soils can reduce N_2_O emissions by more than 90% (**Figure 6**). Therefore, optimizing the timing and placement of NIs, such as mixing them with tillering or ear fertilizers and applying them near the root zone, can significantly enhance their effectiveness in mitigating N_2_O emissions. However, despite the availability of commercial fertilizers pre-mixed with NIs, their high cost and the widespread preference among farmers for traditional fertilizers have hindered their adoption.

It is important to note that the efficacy of NIs is influenced by the interplay of soil environmental conditions and the characteristics of the inhibitors themselves. For instance, while NIs reduce nitrification and denitrification rates, they may inadvertently exacerbate ammonia (NH_3_) volatilization. Combining NIs with urease inhibitors may provide a more effective strategy for regulating urea transformation and achieving greater reductions in N_2_O emissions. Further research is needed to refine the application of NIs and explore their interactions with other management practices in paddy ecosystems.

## 5 Conclusions

This study effectively illuminates the significant role of the rice rhizosphere in modulating N_2_O emissions during different growth phases of rice, as investigated through both pot and incubation experiments. The reduction in N_2_O emissions is predominantly attributed to the interaction of rice root exudates with a higher density of ammonia-oxidizing archaea (AOA) and bacteria (AOB) within the rhizosphere. This association effectively curtails nitrification processes during various rice growth stages. The findings underscore the efficacy of the rhizosphere in mitigating greenhouse gas emissions from paddy soils. Consequently, the application of nitrification inhibitors, particularly those of biological origin, emerges as a promising, environmentally friendly strategy to decrease nitrogen fertilizer loss in rice paddy fields, thereby enhancing sustainability in agricultural practices.

## Data Availability

Datasets are available on request. The raw data supporting the conclusions of this article will be made available by the authors, without undue reservation. Requests to access the datasets should be directed to hpzhang@yzu.edu.cn.
