# Supplementary material for: Soil properties and rhizosphere interactions affecting nitrous oxide emissions with mitigation by nitrification inhibitors in rice growth stages
Source: Front Plant Sci. 2025 Feb 21;16:1501410. doi: 10.3389/fpls.2025.1501410 (PMC11885122; doi:10.3389/fpls.2025.1501410)
Supplement: Supplementary file 1 [file Table1.docx]

**Supplementary Material**

**Soil properties and rhizosphere interactions modulating nitrous oxide emissions in rice growth stages**

Haipeng Zhang, Yiyin Lu, Wanyi Li, Fuxing Liao, Juanjuan Wang, Hongcheng Zhang, Yanju Yang *

*Key Laboratory of Cultivated Land Quality Monitoring and Evaluation (Yangzhou University), Ministry of Agriculture and Rural Affairs/ Co-Innovation Center for Modern Production Technology of Grain Crops, Research Institute of Rice Industrial Engineering Technology, Yangzhou University, Yangzhou 225009, China*

***Corresponding author: Tel: +86 514 8797 9528; Fax: +86 514 8797 8626.**

**E-mail: yangyanju@yzu.edu.cn (Y.J. Yang)**

The Supplementary Material file contains one table.

Table S1 Characteristics of paddy soils.

| Soil | Location | Stage | pH | EC  mS cm^-1^ | AP  mg kg^-1^ | AK  mg kg^-1^ | AN  mg kg^-1^ | TN  g kg^-1^ | TP  g kg^-1^ | OM  g kg^-1^ | NH_4_^+^-N  mg kg^-1^ | NO_3_^-^-N  mg kg^-1^ | C/N | C/P | N/P | NNR |
| --- | --- | --- | --- | --- | --- | --- | --- | --- | --- | --- | --- | --- | --- | --- | --- | --- |
| JR | Rhizosphere | Tillering | 5.94±0.10 | 0.18±0.00 | 9.16±0.00 | 50.99±0.99 | 91.93±1.61 | 1.13±0.01 | 0.46±0.01 | 13.56±0.00 | 6.61±0.07 | 16.94±0.19 | 6.99±0.03 | 17.16±0.30 | 2.46±0.03 | 3.57±0.09 |
|  |  | Jointing | 5.77±0.02 | 0.18±0.00 | 10.86±0.11 | 43.60±0.99 | 101.8±0.00 | 1.03±0.02 | 0.45±0.01 | 13.79±0.16 | 7.37±0.08 | 12.67±0.13 | 7.79±0.25 | 17.88±0.55 | 2.30±0.00 | 3.46±0.37 |
|  |  | Heading | 5.78±0.03 | 0.15±0.00 | 9.95±0.34 | 43.03±0.00 | 97.64±0.89 | 1.04±0.04 | 0.45±0.01 | 14.87±0.17 | 9.23±0.29 | 4.02±0.13 | 8.33±0.46 | 19.32±0.82 | 2.32±0.03 | 3.23±0.16 |
|  |  | Grain-filling | 5.71±0.03 | 0.24±0.00 | 12.23±0.11 | 50.00±0.00 | 142.6±0.89 | 1.09±0.03 | 0.48±0.00 | 15.20±0.15 | 11.11±0.37 | 2.98±0.10 | 8.12±0.28 | 18.32±0.28 | 2.27±0.04 | 2.54±0.16 |
| JR | Non-rhizosphere | Tillering | 5.98±0.06 | 0.39±0.00 | 38.93±0.23 | 69.84±0.00 | 89.07±0.54 | 1.05±0.01 | 0.46±0.01 | 12.91±0.27 | 7.07±0.17 | 13.61±0.32 | 7.15±0.23 | 16.35±0.60 | 2.29±0.01 | 1.14±0.02 |
|  |  | Jointing | 5.91±0.01 | 0.25±0.00 | 35.41±0.34 | 71.83±0.00 | 89.07±0.89 | 1.08±0.07 | 0.48±0.00 | 13.01±0.94 | 11.90±0.17 | 8.33±0.12 | 6.98±0.05 | 15.82±1.11 | 2.27±0.14 | 2.23±0.16 |
|  |  | Heading | 5.89±0.06 | 0.27±0.01 | 46.09±0.34 | 68.85±0.99 | 93.18±1.43 | 1.09±0.00 | 0.47±0.01 | 14.34±0.06 | 11.64±0.39 | 3.42±0.11 | 7.63±0.02 | 17.61±0.50 | 2.31±0.06 | 2.47±0.27 |
|  |  | Grain-filling | 5.84±0.13 | 0.27±0.02 | 42.65±0.36 | 109.52±1.98 | 101.0±2.50 | 0.99±0.04 | 0.45±0.00 | 14.90±0.13 | 12.74±0.12 | 4.17±0.04 | 8.75±0.47 | 19.19±0.15 | 2.20±0.10 | 2.04±0.04 |
| YC | Rhizosphere | Tillering | 7.99±0.06 | 0.23±0.00 | 10.64±0.11 | 51.98±1.98 | 95.14±0.54 | 1.35±0.01 | 0.92±0.01 | 17.71±0.00 | 3.68±0.14 | 23.13±0.88 | 7.63±0.05 | 11.20±0.07 | 1.47±0.02 | 6.69±0.08 |
|  |  | Jointing | 8.02±0.04 | 0.18±0.00 | 10.98±0.00 | 48.02±0.00 | 101.75±0.71 | 1.18±0.04 | 0.90±0.00 | 17.12±0.05 | 3.82±0.09 | 13.61±0.33 | 8.44±0.29 | 11.08±0.08 | 1.31±0.04 | 6.50±0.18 |
|  |  | Heading | 7.92±0.10 | 0.21±0.01 | 10.52±0.00 | 50.99±0.99 | 93.89±3.57 | 1.21±0.03 | 0.87±0.00 | 16.65±0.32 | 4.20±0.06 | 7.52±0.11 | 7.98±0.36 | 11.14±0.18 | 1.40±0.04 | 6.29±0.10 |
|  |  | Grain-filling | 7.63±0.05 | 0.25±0.01 | 10.52±0.45 | 67.86±0.00 | 105.32±0.00 | 1.13±0.05 | 0.87±0.01 | 17.50±0.17 | 4.29±0.13 | 6.18±0.19 | 9.00±0.45 | 11.64±0.23 | 1.30±0.04 | 5.84±0.26 |
| YC | Non-rhizosphere | Tillering | 8.05±0.08 | 0.26±0.00 | 36.20±0.68 | 65.87±0.00 | 93.18±0.71 | 1.22±0.07 | 0.90±0.01 | 16.72±0.20 | 6.13±0.10 | 25.45±0.43 | 7.99±0.36 | 10.82±0.23 | 1.36±0.09 | 5.26±0.10 |
|  |  | Jointing | 7.94±0.05 | 0.27±0.00 | 48.02±0.23 | 68.85±0.99 | 94.61±0.00 | 1.24±0.00 | 0.91±0.00 | 16.82±0.06 | 4.29±0.15 | 15.67±0.55 | 7.87±0.02 | 10.73±0.00 | 1.36±0.00 | 5.29±0.29 |
|  |  | Heading | 8.01±0.06 | 0.25±0.01 | 22.47±0.18 | 65.87±0.00 | 88.36±3.75 | 1.18±0.03 | 0.88±0.01 | 15.78±0.37 | 4.38±0.05 | 8.72±0.09 | 7.75±0.38 | 10.35±0.17 | 1.34±0.04 | 4.89±0.20 |
|  |  | Grain-filling | 7.86±0.07 | 0.32±0.01 | 39.56±0.55 | 91.67±1.98 | 106.21±3.75 | 1.19±0.05 | 0.88±0.01 | 17.75±0.47 | 4.29±0.10 | 7.03±0.16 | 8.65±0.14 | 11.69±0.38 | 1.35±0.07 | 5.09±0.12 |
| *p* value | | Soil | 0.000 | 0.063 | 0.000 | 0.678 | 0.000 | 0.000 | 0.000 | 0.000 | 0.000 | 0.000 | 0.000 | 0.000 | 0.000 | 0.000 |
|  |  | Location | 0.000 | 0.000 | 0.000 | 0.000 | 0.000 | 0.248 | 0.011 | 0.000 | 0.000 | 0.000 | 0.030 | 0.000 | 0.019 | 0.000 |
|  |  | Stage | 0.000 | 0.000 | 0.000 | 0.000 | 0.000 | 0.000 | 0.000 | 0.000 | 0.000 | 0.000 | 0.000 | 0.000 | 0.000 | 0.000 |
|  |  | Soil*Location | 0.316 | 0.000 | 0.000 | 0.000 | 0.000 | 0.692 | 0.732 | 0.632 | 0.000 | 0.000 | 0.901 | 0.038 | 0.128 | 0.637 |
|  |  | Soil*Stage | 0.004 | 0.000 | 0.000 | 0.017 | 0.000 | 0.116 | 0.000 | 0.000 | 0.000 | 0.000 | 0.002 | 0.000 | 0.737 | 0.000 |
|  |  | Stages*Location | 0.017 | 0.000 | 0.000 | 0.000 | 0.000 | 0.000 | 0.000 | 0.022 | 0.000 | 0.000 | 0.001 | 0.000 | 0.018 | 0.000 |
|  |  | Soil*Location*Stage | 0.018 | 0.000 | 0.000 | 0.000 | 0.000 | 0.002 | 0.000 | 0.156 | 0.000 | 0.000 | 0.019 | 0.018 | 0.342 | 0.000 |

Note: Soils JR and YC were collected from Jurong and Yancheng, Jiangsu province, respectively. pH, pounds Hydrogen; EC, electrical conductivity; AP, available phosphorus; AK, available potassium; AN, alkaline nitrogen; TN, total nitrogen content; TP, total phosphorus; OM, soil organic matter; C/N, Carbon and nitrogen ratio; NH_4_^+^-N, ammonium nitrogen; NO_3_^-^-N, nitrate nitrogen; C/N, organic carbon to total nitrogen ratio; C/P, organic carbon to total phosphorus ratio; N/P, total nitrogen to total phosphorus ratio; NNR, net nitrification rate. Multi-factor ananlysis of variance *p* values for soils, location, stages and there interactions are shown at the bottom of the table.
